# Conservation of Genomic Information in Multiple Displacement Amplified Low-Quantity Metagenomic Material from Marine Invertebrates

**DOI:** 10.3390/md21030165

**Published:** 2023-03-02

**Authors:** Andrea Iselin Elvheim, Chun Li, Bjarne Landfald

**Affiliations:** Faculty of Biosciences, Fisheries and Economics, Norwegian College of Fishery Science, UiT The Arctic University of Norway, NO-9037 Tromsø, Norway

**Keywords:** marine invertebrates, microbiomes, multiple displacement amplification, metagenomics, biosynthetic gene clusters

## Abstract

Marine invertebrate microbiomes have been a rich source of bioactive compounds and interesting genomic features. In cases where the achievable amounts of metagenomic DNA are too low for direct sequencing, multiple displacement amplification (MDA) can be used for whole genome amplification. However, MDA has known limitations which can affect the quality of the resulting genomes and metagenomes. In this study, we evaluated the conservation of biosynthetic gene clusters (BGCs) and enzymes in MDA products from low numbers of prokaryotic cells (estimated 2–850). Marine invertebrate microbiomes collected from Arctic and sub-Arctic areas served as source material. The cells were separated from the host tissue, lysed, and directly subjected to MDA. The MDA products were sequenced by Illumina sequencing. Corresponding numbers of bacteria from a set of three reference strains were treated the same way. The study demonstrated that useful information on taxonomic, BGC, and enzyme diversities was obtainable from such marginal quantities of metagenomic material. Although high levels of assembly fragmentation resulted in most BGCs being incomplete, we conclude that this genome mining approach has the potential to reveal interesting BGCs and genes from hard-to-reach biological sources.

## 1. Introduction

In the last two decades, high throughput metagenomic sequencing projects have generated comprehensive insights into the genetic diversities of microbiomes, including their commonly large proportion of hitherto uncultivated phylotypes. Main efforts have been on elucidating phylogenetic breadth and metabolic repertoires in environmental contexts, but metagenomic data have also provided the basis for more applied approaches, including searches for genes of individual enzymes or complete secondary metabolite synthesis pathways with possible industrial and health-related potentials [1,2,3,4,5].

Limitations on available microbial DNA are possible hurdles for metagenomic approaches, especially when studying typical low-biomass environments or low abundance microbiomes, such as clean rooms and air [6,7] ecological micro-niches [8], and single specimens of small organisms or small tissue samples [9,10]. The same problem may arise when the microbial cells are separated from the host tissue, if the host organisms have low densities of associated microbes or the process has a particularly low yield. Individual variations in microbiome composition or natural variations between microenvironments might also be obscured if combining the microbiomes of several individuals or increasing sample volumes from microbial communities [11]. For rare and endangered species and in vulnerable environments, collection should also be minimised for reasons of environmental preservation [12,13].

As an alternative to collecting a larger amount of sample material, DNA can be amplified prior to sequencing. The most commonly used method is multiple displacement amplification (MDA), where the phi29 polymerase is employed for isothermal whole genome amplification (WGA). The phi29 polymerase is robust against chemical contaminants [14,15,16], and, as demonstrated by its use in WGA of single bacterial cells, even femtogram DNA quantities may suffice as a template for amplification [15,17]. However, with low amounts of input material, DNA contaminations are a major concern [18]. There are reported shortcomings of MDA, mainly related to uneven amplification (amplification bias) and chimaera formation [19]. Non-uniform read coverage and potential intra- and intergenomic chimaeras add an extra challenge to the genome assembly of MDA-amplified genomes or metagenomes [20]. The amplification bias also distorts quantitative aspects of metagenome studies [6,21,22]. Other approaches for WGA exist, a notable example being multiple annealing and looping-based amplification cycles (MALBAC) [23]. MALBAC has been reported to improve on certain aspects of MDA, but it comes with its own set of shortcomings [24]. 

The present study addresses the feasibility of employing MDA-amplified metagenomic DNA as a basis for disclosing novel genetic features in collections of bacteria too small for straightforward metagenomic sequencing. The source material was invertebrate tissue from hard-to-access Arctic seafloor locations. Marine invertebrate microbiomes, of sponges in particular, have been a rich source of bioactive compounds and biocatalysts [25,26,27,28]. Benthic invertebrates are routinely collected from the seafloor for bioprospecting purposes with tools like dredges and scrapers. Such techniques frequently result in mere fragments of organisms, with correspondingly lowered quantities of associated microorganisms. Evidently, these microbiomes may still be interesting in a bioprospecting context due to their unique geographical or taxonomic origin. Hence, we explored if MDA-generated DNA was still a useful starting point for metagenomic exploration. The conservation of sequence information on biosynthetic gene clusters (BGCs), such as non-ribosomal peptide synthetases (NRPSs), ribosomally synthesised and post-translationally modified peptides (RiPPs), and polyketide synthases (PKSs), and of individual enzymes was estimated. The study of the initial fragment material was extended by including dilutions from more bacteria-rich preparations and three reference bacteria species.

## 2. Results

### 2.1. Study of Model Strains

MDA was run on samples of three strains of bacteria with established whole-genome sequence information, both separately and as mixtures. The MDA reaction mixtures were estimated to contain three cells for the individual strains, and ten cells in total, with equal densities of each strain in the mixed samples. After MDA, replicate samples were picked for Illumina sequencing based on results from Sanger sequencing of PCR amplicons of the 16S rRNA gene. MDA products were discarded if no PCR products were produced or the sequences did not match the expected source strain. For *Bacillus subtilis* and *Escherichia coli*, two out of eight samples each were found satisfactory for Illumina. For *Vibrio atlanticus*, six out of eight samples were picked, as were all eight mixed samples. The resulting assemblies were fragmented, with many short contigs (Table 1 and Table 2). Three of the sequenced *V. atlanticus* samples, VA2, VA4 and VA5, that showed low 16S rRNA gene sequence quality also showed lower quality assemblies after the Illumina sequencing. VA4 stood out negatively with a very low coverage of the reference genome and short average contig length. For the remaining single-strain samples, the assemblies covered between 59 and 90% of the reference genomes, and the unaligned fractions were less than 5%. 

The covered genome fractions for the mixed samples were above 91% for *E. coli* in all assemblies, except M4, where it was down to 84%. For *B. subtilis* and *V. atlanticus*, the covered genome fractions were noticeably smaller and more variable. The unaligned fraction was less than 2% for all samples. The mean sequencing depth and its standard deviation were higher for samples with single strains compared to the mixed samples. For all samples, the standard deviation was higher than the mean, showing that the sequencing depths were highly variable. This was likely an effect of MDA bias [19].

**Figure 1:**
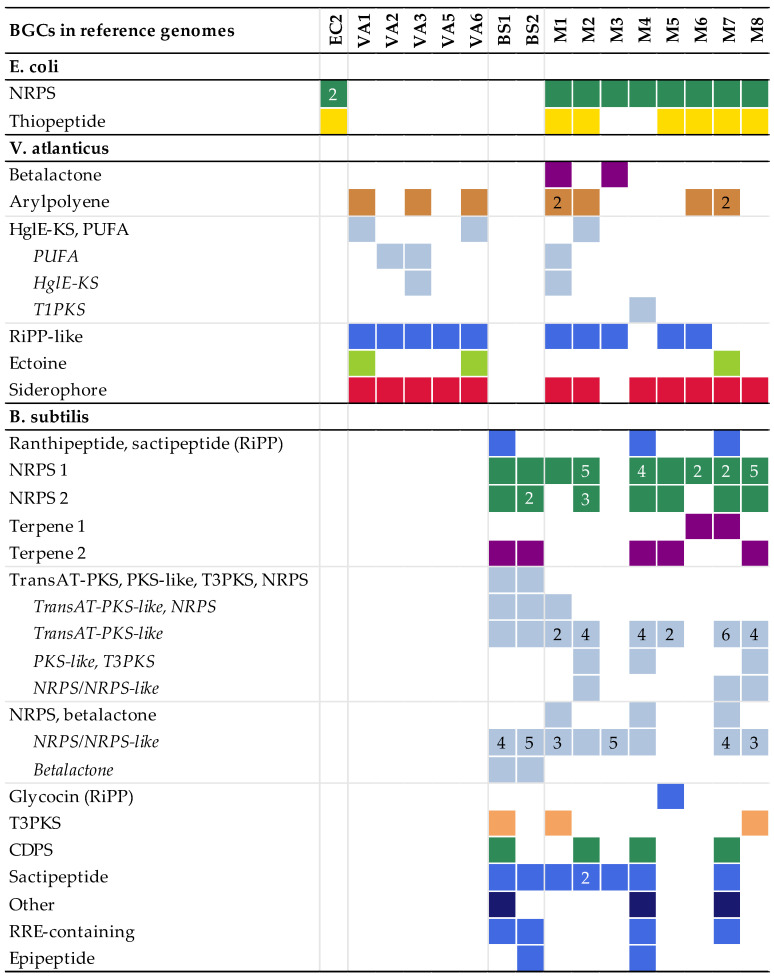
The number and types of biosynthetic gene clusters (BGCs) detected in the multiple displacement amplification (MDA) assemblies mapped to the corresponding BGCs present in the reference genomes. More than one fragment mapping to the same reference BGC is indicated by the number. Hybrid BGCs fragmented into different combinations of BGC types are listed as every variant detected. The colours refer to the BGC type. Colours, numbers and types of BGCs are reported according to antiSMASH. No BGCs were detected in EC1 and VA4; these assemblies are therefore excluded from the figure. Abbreviations: non-ribosomal peptide synthetases (NRPSs), polyketide synthases (PKSs), heterocyst glycolipid synthase-like PKS (HglE-KS), polyunsaturated fatty acids (PUFA), type I PKS (T1PKS), ribosomally synthesised and post-translationally modified peptides (RiPPs), trans-acyltransferase PKS (transAT-PKS), type II PKS (T3PKS), tRNA-dependent cyclodipeptide synthases (CDPS), RiPP recognition element (RRE).

The BGCs found by antiSMASH in the model experiment assemblies were fragmented, as compared to the contiguous BGCs of the reference genomes. The more complex BGCs, such as NRPS clusters and a large NRPS-PKS hybrid cluster in *B. subtilis*, were particularly fragmented. Some clusters were not found in all replicate assemblies (Figure 1).

### 2.2. Study of Marine Invertebrate Microbiome Samples

#### 2.2.1. Taxonomy of Source Material

One invertebrate was identified as the bryozoa *Alcyonidium gelatinosum* according to established morphological criteria, while five sponge samples were tentatively identified by a DNA barcoding approach (Table 3). One sample (sample B) showed identical best hits at the species level for the 28S rRNA and CO1 gene sequences, with sequence identities of 98.3% (28S rRNA gene) and 99.6% (CO1 gene). The remaining four sponge samples gave lower identities with current database entries (89−97%) and divergent “best hits” at species, genus, or family level between the two genes employed in the present study.

#### 2.2.2. Sequencing and Assembly of Marine Invertebrate MDA Amplified Metagenomes

All assemblies generated from the marine invertebrate microbiomes were heavily fragmented (Table 4), likely caused by a combined effect of the MDA reaction, short-read sequencing, and in the cases of samples with higher diversity, too low sequencing depth. The total lengths of the assemblies generally followed the estimated number of cells used as an MDA template, with the samples estimated to contain lower numbers of cells having shorter assemblies. The exceptions were samples E3 and F1. As the length of assemblies varied while the number of sequencing reads was more stable, the sequencing depths were higher for samples with shorter assemblies. However, the depth had a higher variation. 

#### 2.2.3. Taxonomic Profiles

**Figure 2:**
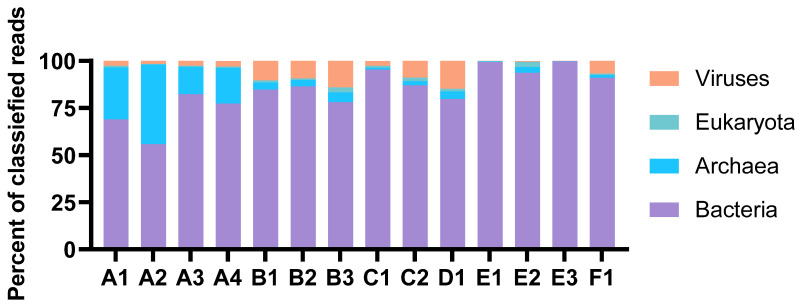
Distribution of reads classified at the domain level. The abundances are shown as per cent of classified reads. The figure is based on results from Kaiju in Kbase (NCBI BLAST nr+euk database).

**Figure 3:**
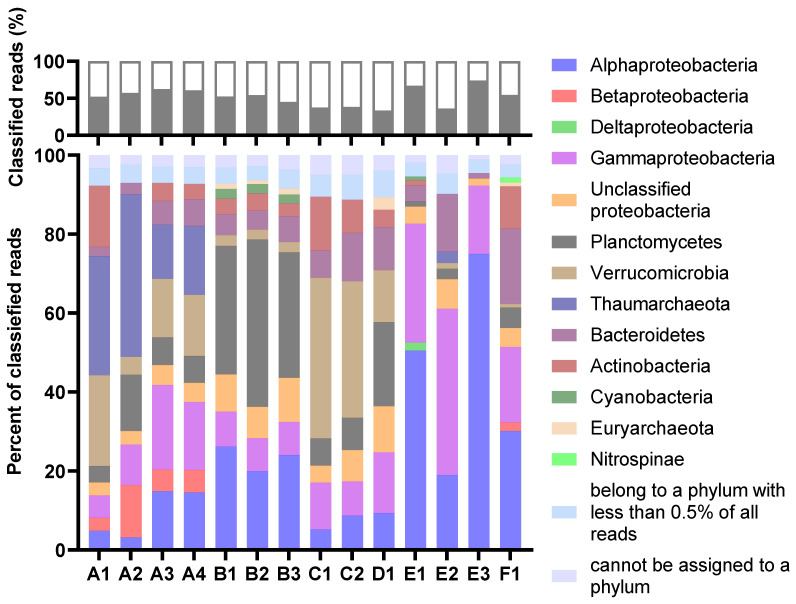
The per cent of reads classified at the phylum level (top) and the distribution of the classified reads at the phylum or class (Proteobacteria) level (bottom). For the lower graph, the abundances are relative, shown as a per cent of classified reads. The legend refers to the lower graph. The figure is based on results from Kaiju (MarDB).

The taxonomic profiles were consistent for MDA amplicons of the same animal origin, with only minor variations in relative abundances. The percentage of reads classified as eukaryotic was very low in all samples (Figure 2). Thus, the process of separating bacterial cells from the invertebrate host tissue and removal of extracellular DNA was considered successful. The Proteobacteria were the most abundant prokaryotic phylum in total (Figure 3). Their dominance was most pronounced in sample E, where more than 70 per cent of reads from all three MDA products were categorised as having proteobacterial origin. The Planctomycetes were a common phylum in assemblies originating from B and D, while Verrucomicrobia were particularly abundant in the A and C assemblies. Archaeal reads were almost exclusively assigned to the Nitrosopumilaceae family of Thaumarchaeota. This ammonia-oxidizing group was found to be abundant in sample A. According to the sample A distributions, the abundances of each taxon seemed more variable for samples with higher levels of dilutions before MDA. The A3 and A4 distributions were almost identical, while A1 and A2 showed a marked difference.

#### 2.2.4. Annotation of Genes and Enzymes

**Figure 4:**
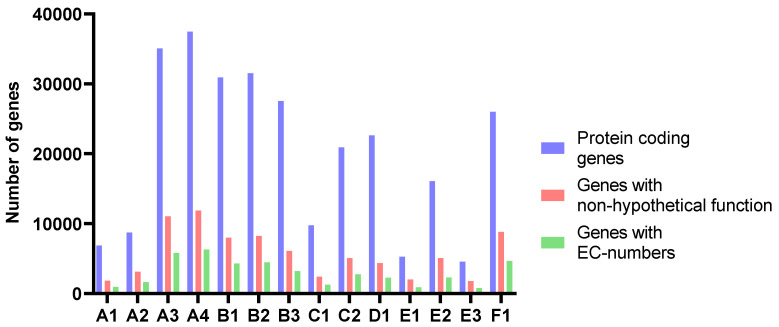
The number of protein-coding genes, genes with non-hypothetical function, and genes with EC-numbers predicted and annotated by Prokka.

**Figure 5:**
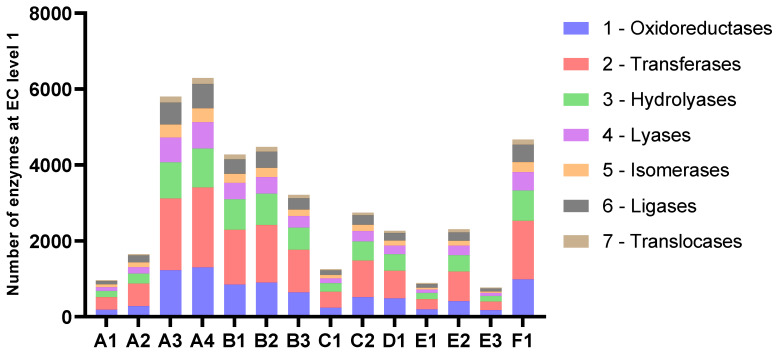
Number of enzymes categorised by EC-numbers at level 1.

The number of predicted genes followed the assembly length for all samples (Figure 4 and Table 4). The average length of proteins ranged from 204 to 235 amino acids. The amounts of genes annotated as enzymes with EC-numbers varied in accordance with the number of predicted genes in the assemblies and the length of the assemblies. At level 1, the relative distribution of EC-numbers was very similar for all samples (Figure 5). Transferases were most abundant, followed by oxidoreductases and hydrolyases. 

Table 5 shows the number of genes annotated to a selected group of enzymes with potential biotechnological relevance. This includes various hydrolases and nucleic acid-modifying enzymes. The assemblies of sample B contained several genes annotated as glycosidases (EC 3.2.1), which were not found in the other samples. BLASTp searches showed nearly all the sample B glycosidase sequences to be affiliated with the phylum Planctomycetes, to the families Planctomycetaceae or Pirellulaceae. This is consistent with the results from the taxonomic analyses, which showed the reads classified as Planctomycetes in B to be assigned to these two families. Several of the potential glucosidases were likely to be truncated as they were shorter than their hits in the database or shorter than aligned genes from the other two replicate B assemblies, while others had the same or similar length as their database matches. 

#### 2.2.5. Biosynthetic Gene Clusters

**Figure 6:**
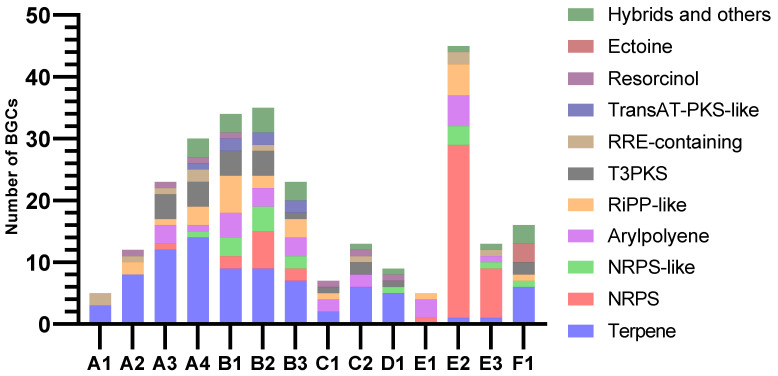
Numbers and classifications of BGCs as identified by antiSMASH v.6.1.1.

AntiSMASH predicted similar patterns of BGC class distribution in assemblies of the same animal origin, while the variation between the animal samples was more pronounced (Figure 6). Almost all clusters extended to one or both contig edges, making it likely that many were incomplete. The overall most common BGC class was terpene, which was identified in all samples except E1. Only A4 contained one complete terpene BGC, while all others extended too close to one or both contig edges to conclude on completeness. The core terpene biosynthetic genes likely originated mainly from Planctomycetes, Verrucomicrobia, and Betaproteobacteria. Only ten of the terpenes had matches in KnownClusterBlast in antiSMASH, nine of these matching carotenoids from various phyla. The last one showed similarity to a hopene-encoding BGC in *Streptomyces coelicolor*. 

Assemblies from samples B and E stood out with large proportions of BGCs categorised as NRPS or NRPS-like. Assemblies from sample B also had many BGCs with PKS domains. Due to their heavy fragmentation, many of the predicted NRPS clusters, PKS clusters, and NRPS-PKS hybrids were assumed to be fragments of the same BGCs. Type III PKSs (T3PKSs) were also found in assemblies from all invertebrate samples except E. Most PKS type III BGC sequences were affiliated with Actinomycetia, Planctomycetes and Verrucomicrobia. The B1 and C2 assemblies both had one T3PKS BGC from Actinobacteria that resembled an alkylresorcinol BGC from *Streptomyces griseus* in the MIBiG database. These clusters were potentially complete, based on the length and number of genes of the BGCs from the MIBIG comparison and KnownClusterBlast hits. The T3PKS genes of the two samples were 96% similar to each other and approximately 96% similar to their best BLASTP hits (nr database). Their best hits were from an uncharacterised Actinomycetes bacterium. The assemblies from sample B shared one potentially complete T3PKS cluster from Planctomycetes. It had low similarities to clusters in MIBIG and no matches in KnownClusterBlast.

Samples B and E also contained a high number of RiPP-like BGCs. A lanthipeptide class II (RiPP) BGC was predicted in A4. This BGC was best assembled by metaSPAdes. The cluster contained *lanM*, responsible for the lanthipeptide modification, a peptidase, a peptide ABC transporter, and three predicted precursor peptide candidates. The BGC extended to both contig edges but seemed to contain the essential genes, so it could potentially be complete. The closest hit by antiSMASH ClusterBlast was from *Candidatus* Methylopumilus planktonicus (NZ_LN827929.1). It had a similar organisation of genes but different predicted precursor peptides. The cluster had no KnownClusterBlast hits. The closest BLASTP match for the predicted *lanM* gene was from an unclassified Nitrosomonadales strain (MBT3826530.1), with 88.30% identity and the same gene length.

## 3. Discussion

To the best of our knowledge, very few studies have reported on the use of MDA products generated from bacterial community DNA for whole genome sequencing and assembly of metagenomic DNA. MDA is routinely used after single cell sorting [29], where the input amounts, but also the complexity, is lower than for metagenomes. Within bioprospecting, metagenomic MDA products have been used directly to make functional expression libraries [9,30,31]. Most previous studies on sequencing metagenomic MDA products have performed amplicon sequencing of 16S rRNA gene regions to study community profiles [12,32] or direct analysis of the sequencing reads without prior DNA assembly [13,22,24]. A few studies assemble mini-metagenomes after fluorescence in situ hybridisation (FISH) combined with fluorescence-activated cell sorting (FACS), with a focus on developing methods for sorting and characterising specific taxonomic groups [33,34]. They report fragmented assemblies, as is characteristic after MDA. Thus, many advantages and limitations of using MDA for DNA amplification are well documented. The present study focused specifically on the feasibility of applying MDA for genome mining of low-quantity marine invertebrate microbiomes.

The present study, including six microbiome samples and three reference strains, was too limited to give a general estimate of the outcome of the employed methodological approach. The metagenomic material solely originated from marine invertebrates, principally sponges. On the other hand, the DNA templates for the MDA were all based on purified prokaryotic cell preparations. Hence, the nature of the source material was not expected to influence significantly on the quality of the MDA products. If such purified cell material is achievable, our data, therefore, suggest that extensive sequence information on BGCs and enzyme genes was obtainable in MDA products generated from marginal quantities of metagenomic DNA. This conclusion was substantiated by the observation that all known BGCs in the reference strain genomes were identified in at least one replicate MDA product made from mixtures of the reference strains. In contrast to the model experiment, a wide variation in relative abundances of taxonomic groups must be expected in the marine invertebrate microbiotas. Hence, we anticipated that BGCs and other genomic elements of the metagenomic MDA products chiefly originated from the dominant phylogroups of the hosts’ microbiota. In the MDA templates based on very low numbers of cells, statistical randomness was an additional factor contributing to biased taxonomic representation in the amplified metagenomes. 

There were additional methodological limitations to the applied approach. The Illumina-sequenced MDA products resulted in fragmented assemblies and mainly incomplete BGC sequences. The extensive fragmentation was likely caused by MDA bias [10,33,34], combined with the effects of limitations of the chosen sequencing technology and low sequencing depth in the samples with the largest taxonomical variety. Very few of the BGCs were categorised as complete by antiSMASH. However, a few small, less complex BGCs appeared complete but not sufficiently far from the contig edge to satisfy the criteria of antiSMASH [35]. Especially NRPS and PKS clusters are known to be fragmented in assemblies based on short-read sequencing because of their size and repetitive regions [36,37]. This pattern was manifested in the assemblies of reference bacterial strains, where NRPS and PKS BGCs were present as one or more fragments, and the detected number of BGCs with NRPS and PKS modules was an overestimation compared to the number of such BGCs detected in the reference genomes. Despite the limitations, we conclude that the marine invertebrate microbiome MDA products gave useful information on the types of BGCs and enzyme genes that were present. This gives a solid basis for further genome mining efforts through the improvement of the assemblies to recover complete BGCs of interest. 

Long-read sequencing technologies, like Oxford Nanopore and PacBio SMRT, are often used in combination with Illumina sequencing to improve contig length by helping assemble hard-to-solve regions, like NRPS and PKS BGCs [37,38]. However, the DNA fragments synthesised by MDA are typically around 12 kb on average, up to 100 kb [39]. This would limit the read lengths that could be achieved, even by long-read sequencing. According to the producer, Nanopore sequencing of MDA products results in read lengths mostly up to 5 kb [40]. Thus, the assembly improvement of MDA products by using long-read sequencing is expected to be limited. In a recent study of a *H. panicea* holobiont, Illumina and Nanopore reads were combined to form a slightly improved but still heavily fragmented hybrid assembly. Here, two out of three rounds of Nanopore sequencing were performed on MDA-amplified DNA. However, the authors also reported high levels of degradation already after DNA extraction, which they hypothesised were due to degradation pathways in either the sponge itself or the sponge microbiome [41]. This would add to the negative effect on read length caused by MDA. An additional complicating effect is that longer reads increase the probability of capturing the chimaeric sequences occasionally created during the MDA reaction [42]. 

The average sequencing coverage varied from 5.6× to 107×; thus, many of the larger assemblies could probably be improved by gaining higher sequencing coverage. The total lengths of the generated assemblies were expected to follow the number of cells used as the MDA template, as reduced numbers of cells would mean lowered taxonomic and genomic variation. While this was true for most samples, there were exceptions, possibly caused by some difficulty in accurately estimating the bacterial concentrations in the prokaryotic cell preparations and the random variation caused by diluting cells to low numbers. The bacterial counts in five of our MDA templates were estimated to be less than ten, and the amplification products mostly had correspondingly short assemblies and high sequencing coverages. However, the read coverages were very uneven, which pointed to the effects of MDA bias as the main cause of fragmentation. Previous studies have shown that low amounts of template cause increased amplification bias, which leads to more uneven read coverage and possible allelic dropout [19]. 

As the microbial preparations in this study have undergone separation from the host tissue, dilution, and MDA, the community profiles were expected to deviate from the original marine invertebrate microbiomes. In studies of marine invertebrate metagenomes, DNA is either extracted directly from the host animal tissue, implying that genomic material from both host and associated microorganisms are included (hologenomics) [41,43], or the microbial cells are separated from the host tissue before sequencing [44,45,46]. We employed the latter approach, as bacteria-sized cells were separated from their marine invertebrate host tissue prior to MDA. By avoiding the host DNA in the sequencing process, the yields of microbial sequences were increased correspondingly. However, the process of separating the prokaryotic cells from the host tissue might have disturbed the taxonomic profiles of the microbiomes [30], and some loss of marginally represented bacterial groups at this stage cannot be excluded. 

Depending on the number of microbes present and the yield of microbial cells after separation from the host tissue, the amount of DNA might be too low for direct sequencing as a consequence of the procedure as such. Subsequent processing of the prokaryotic preparations, i.e., targeting specific taxonomical groups, morphologies, or genetic features, will reduce the amount of template material even more. Thus, MDA has also been used in studies of mini-metagenomes after FACS-sorting [33,34]. Such approaches are particularly relevant in a bioprospecting context, as they might increase the chances of novel discoveries by targeting less studied groups instead of the most abundant strains. 

In sample B, Planctomycetes was the second most abundant phylum, with 20–30% of reads assigned to it. Such relative abundances of Planctomycetes were not consistent with results from other studies of *H. panicea*, where Planctomycetes were shown to constitute less than 5% of classified reads [11,47,48,49,50]. Our study gave no possibility to clarify if the difference was related to actual biological variations, the process of separating the prokaryotic cells from the host tissue, or MDA bias. Certain phyla are known for having higher biosynthetic potentials, Planctomycetes being one of them [51,52,53], and many of the BGCs in the B assemblies seemed to originate from Planctomycetes. The assemblies from sample B, together with assembly E2, represented the highest number and widest variety of BGCs detected by antiSMASH. They contained many NRPS or NRPS-like BGCs. Assemblies from sample B also contained BGCs with PKS domains, categorised as both trans-acyltransferase-PKS-like (transAT-PKS-like) and type I PKS, in addition to several type III PKS BGCs. Most of the BGCs had low or no similarities to known clusters and could therefore be worth further studies, either for discovering new compounds or for connecting known compounds to their genetic origin.

## 4. Materials and Methods

### 4.1. Sampling and Sample Preparations

Seafloor invertebrate samples were collected by Agassiz trawl or triangular dredge on a research cruise with RV Helmer Hansen in August 2016. Samples A, C, D and F originate from the eastern coastal regions of the Svalbard archipelago (between 79°12′ and 80°7′ N, depth range 75–177 m), while sample B was collected off the coast of northern Norway (71°8′ N, depth 73 m) and sample E in the central Barents Sea (74°33′ N, depth 107 m). On board the ship, the samples were washed in sterile NaCl (2%). When feasible, the outer layers of the animal tissue were removed aseptically. The remaining tissue was manually disintegrated in 2% NaCl in Stomacher® sterile plastic bags. The resulting fluid phase was separated from the solid remains, added glycerol to a final concentration of 15% (*w*/*v*), and stored at −25 °C. For extended laboratory storage, the samples were kept at −80 °C. 

#### Taxonomic Identification of Sponge Source Material

For inferring the taxonomy of the sponge samples, tissue homogenate glycerol samples were thawed, pelleted at 6000× *g*, and washed twice with NaCl solution (5 M) to remove the glycerol after freeze storage. Depending on the amount of available material, approximately 100 mg of biomass was used for DNA extraction with DNeasy PowerSoil Pro Kit (Qiagen, Hilden, Germany). Sponges were identified by amplifying and sequencing the B10-C1 region of the 28S rDNA [54,55] and a region of the CO1 gene [55,56], using sponge-specific primers Por28S-15F/Por28S-878R [54,55] and dgLCO1490/dgHCO2198 [56]. The PCRs were set up according to the manufacturer’s protocol for Platinum™ II Hot-Start Green PCR Master Mix (2X) (Invitrogen by Thermo Fisher Scientific, Eugene, OR, USA). 

The PCR products were purified using the Bio-On-Magnetic-Beads (BOMB) protocol for clean-up and size exclusion [57]. The magnetic beads used were Sera-Mag™ SpeedBeads™ magnetic carboxylate-modified particles (Cytiva, Marlborough, MA, USA). Beads were pelleted on a magnet from 700 µL of the original stock, washed in TE buffer and resuspended in 1 mL TE before being added to the binding buffer. Cycle sequencing reactions were set up with the BigDye™ Terminator v3.1 Cycle Sequencing Kit (Applied Biosystems™ by Thermo Fisher Scientific, Vilnius, Lithuania), and the samples were sequenced at The University Hospital of North Norway sequencing facility, both forward and reverse directions, targeting both 28S rRNA and CO1 genes. The resulting sequences were quality controlled and subjected to BLASTn (NCBI nr/nt database) default settings. 

### 4.2. Separation of Bacteria-Sized Cells from Homogenized Invertebrate Tissue

Ultrapure water, aqueous solutions, and laboratory devices to be used for handling the purified bacteria preparations were autoclaved at 134 °C for 130 min to disintegrate contaminating DNA. Tissue homogenates (1 g) were thawed, added 750 µL phosphate-buffered saline (PBS) (50 mM, pH 7.4, 2% NaCl) and further disintegrated on ice in a Potter-Elvehjem homogeniser for 3 min. The subsequent steps to separate bacteria from host animal tissue were adjusted to varying rheological properties of the material but generally consisted of repeated 30 min low-speed centrifugations and washings in the centrifuge with PBS. The g-force was increased stepwise from 200× *g* to 1000× *g* in the final centrifugation. The pooled supernatants were filtered through a 12 μm Cyclopore track-etched membrane (Whatman, Cytiva, Marlborough, MA, USA), then centrifuged at 8000× *g* for 20 min to collect material of prokaryotic cell size. The resuspended pellet (PBS) was treated with Plasmid-Safe™ ATP-dependent DNase (Lucigen, Madison, WI, USA) in a total reaction volume of 0.5 mL, according to the manufacturer´s protocol. Centrifugation (8000× *g*) and resuspension in 0.5 mL PBS were repeated twice before final resuspension in 1 mL PBS.

### 4.3. Estimation of Bacteria Concentration

The concentration of bacteria in each sample was estimated by epifluorescence counting [58,59]. The purified bacterial preparations were diluted 1:100 in 4 mL PBS and collected on 0.2 µm black polycarbonate Nucleopore filters (Whatman, Cytiva, Marlborough, Massachusetts). The filters were dried and stained in the dark with SYBR Gold (Molecular Probes, Leiden, The Netherlands) for 15 minutes. The stained filters were mounted with mounting solution (1:1 PBS and glycerol with 0.1% p-phenylenediamine), and the cell numbers were estimated by counting 10–25 random fields in an epifluorescence microscope (Leica DM6000 B).

### 4.4. Multiple Displacement Amplification

Based on the epifluorescence counting, the bacteria preparations were diluted to various degrees before being used as a template for MDA. The estimated number of cells in the reaction mixtures varied from 2 to 850 (Table 4). MDA was performed with the illustra™ Single Cell GenomiPhi DNA Amplification Kit (GE Healthcare, Little Chalfont, UK), according to the manufacturer’s protocol for whole genome amplification of microbial cells, including positive and negative controls. The resulting yields were determined with the Qubit™ dsDNA BR Assay Kit (Applied Biosystems™ by Thermo Fisher Scientific, Eugene, OR, USA). PCR amplification and Sanger sequencing of 16S regions were performed to confirm successful MDA of bacterial DNA and to exclude samples with evident bacterial contaminations. The 16S rDNA regions were amplified with primers B27F/U1492R, and Sanger sequencing was performed as described above. If amplicons did not generate intelligible 16S rRNA gene sequences or the sequences pointed to a non-marine bacterial source, the MDA products were discarded. The MDA products were purified with the DNeasy Blood and Tissue kit (Qiagen, Hilden, Germany) without the steps for lysis in the beginning. The DNA was eluted in EB buffer (Qiagen, Hilden, Germany). The concentration of purified DNA was estimated using Qubit™, and the purity by Nanodrop™.

### 4.5. Illumina MiSeq Sequencing

Sequencing libraries were constructed with the Nextera™ DNA Flex Library Prep kit (Illumina) and barcoded with Nextera™ DNA CD Indexes (Illumina, San Diego, CA USA). Each library was quantified with qPCR (NEBNext® Library Quant Kit for Illumina; New England BioLabs, Ipswich, Massachusetts, USA) to achieve uniform pooling of libraries and optimal cluster density during sequencing. Sequencing was performed on a MiSeq instrument with the MiSeq Reagent kit v2 (500 cycles) (Illumina, San Diego, CA, USA). The sequencing of MDA products from marine invertebrate microbiomes generated from 1.7 to 3.0 million reads. 

### 4.6. Analysis of Sequence Data

Quality control, pre-processing, assembly, and annotation of reads was performed in KBase [60]. Kaiju (v1.7.3) [61] in Kbase was used to characterise raw reads by domain with the NCBI BLAST nr + euk database. Stand-alone Kaiju (v1.7.2) was used to assess the taxonomy of prokaryotes from the raw reads, with MarDB [62] containing marine microbial genomes. Quality control of raw reads was performed with FastQC (v0.11.5) [63]. Low-quality sequences were removed by Trimmomatic v0.36 [64], and overlapping read pairs were merged with ea-utils FASTQ-JOIN v2.0.2 [65]. The operations were performed with KBase standard parameters, except 15 bases head crop by Trimmomatic. Assemblies were created with SPAdes (v3.12.0) [66,67]. Assemblies by standard SPAdes, metaSPAdes and scSPAdes (single cells) were compared. As standard SPAdes gave longer contigs for the majority of samples, these assembly statistics are shown, and further analyses were performed on the standard SPAdes assemblies unless stated otherwise. The assembly statistics were generated by QUAST [68], and the sequencing coverage was assessed by Bowtie2 v2.3.2 [69] by mapping the raw reads back to the assemblies. The assemblies were annotated with Prokka v1.14.5 [70] using the KBase standard parameters. BGCs were predicted using the online version of antiSMASH v6.1.1 [71], with the features KnownClusterBlast, ClusterBlast, SubClusterBlast, MIBIG cluster comparison, ActiveSiteFinder, RREFinder, Cluster Pfam analysis, and Pfam-based GO term annotation enabled. Selected genes from BGCs were, in addition, characterised by comparisons in BLASTp (NCBI nr database). 

### 4.7. Model Experiment

The effects of MDA on simulated mini-metagenomes containing established type strains in defined relative abundances were studied by use of *B. subtilis*, *E. coli*, and *V. atlanticus*. Cultures of *E. coli* and *B. subtilis* were grown overnight in LB medium at room temperature and *V. atlanticus* in FMAP medium [Difco marine broth (15 g), peptone (5 g), agar (15 g), seawater (300 mL), distilled water (700 mL)] at 10 °C. The bacterial samples were diluted 1:100 and 1:1000, and 4 mL was filtered through a 0.2 µm black polycarbonate Nucloeopore filter (Whatman, Cytiva, Marlborough, Massachusetts ). The concentrations of bacteria were estimated by epifluorescence counting after SYBR Gold staining, as described above. Based on these counts, the samples were either diluted to contain an estimated average of 3 cells per MDA reaction or mixed to contain equal densities of each strain and diluted to 10 cells per MDA reaction. MDA was run as described above but with half reaction volumes. Eight samples of each of the single strain cultures and eight mixed samples were amplified.

The MDA products were purified by DNA precipitation, according to the QIAGEN Supplementary Protocol: Purification of DNA amplified using REPLI-g® Kits. PCR amplification and sequencing of the 16S rDNA regions were performed as described above to confirm successful MDA and the identity of the amplified material. In total, 19 samples were chosen for Illumina sequencing, 6 with *V. atlanticus*, 2 each of *E. coli* and *B. subtilis* and all 8 mixed samples. The remaining samples either did not generate any PCR product when using primers targeting 16S rDNA, or they returned non-identifiable sequences after Sanger sequencing. Illumina libraries were prepared and sequenced as described above. The sequencing yielded 1.0 to 1.6 million reads for the single-strain samples and 1.6 to 2.5 million reads for the mixed samples. The analyses of sequencing data were performed as described above for the invertebrate samples, but the assemblies generated by scSPAdes were used for further analyses. The assemblies were compared to reference genomes in QUAST and antiSMASH. The genomes used were *B. subtilis* (NC_000964.3), *E. coli* (NC_000913.3), and *V. atlanticus* (NC_011753.2).

## Figures and Tables

**Table 1:** Assembly statistics for the single-strain samples compared with reference genomes. Assembly statistics are based on contigs of size ≥ 500 bp unless otherwise noted. The sequencing coverage with its standard deviation, the number of predicted protein-coding genes and the average protein length are also shown.

	BS1	BS2	EC1	EC2	VA1	VA2	VA3	VA4	VA5	VA6
Total genome fraction (%)	74.5	59.0	66.7	89.0	83.2	50.3	63.2	2.3	43.1	90.4
Total alignment (kbp)	3491	2907	3553	4536	4176	3106	3267	143	2212	4598
Largest alignment (kbp)	136.0	46.9	126.1	102.6	121.2	65.1	55.2	11.2	18.3	69.5
Unaligned fraction (%)	3.7	4.9	3.9	3.4	2.6	6.1	5.0	51.0	12.6	2.7
Total length (kbp)	3626	3057	3698	4694	4287	3309	3437	292	2531	4724
#Contigs (≥1000 bp)	453	429	332	378	514	541	570	86	665	465
#Contigs (≥10,000 bp)	94	87	84	127	122	83	86	2	18	139
largest contig (kbp)	136.1	60.3	181.0	102.6	121.2	130.0	55.2	13.2	26.3	69.6
N50	15,245	12,593	30,060	30,064	14,895	9276	8952	1914	2604	20,874
Mean sequencing coverage	56.8	93.8	68.1	68.0	60.5	59.2	77.6	493.6	38.1	52.8
Standard deviation	111.9	176.5	99.5	105.9	105.7	140.1	188.2	1346.0	416.2	92.8
Number of protein-coding genes	3520	2988	3464	4377	3659	2883	2988	236	2174	4092
Average protein length (aa)	265	254	284	291	293	271	275	167	221	298

BS: *B. subtilis*; EC: *E. coli*; VT: *V. atlanticus*.

**Table 2:** Assembly statistics for samples containing a mix of *E. coli*, *B. subtilis* and *V. atlanticus* in equal densities (3.3 cells per strain), compared with reference genomes. Assembly statistics are based on contigs of size ≥ 500 bp unless otherwise noted. The sequencing coverage with its standard deviation, the number of predicted protein-coding genes and the average protein length are also shown.

	Mix 1	Mix 2	Mix 3	Mix 4	Mix 5	Mix 6	Mix 7	Mix 8
Total genome fraction (%)	67.6	66.1	50.5	50.9	42.8	39.9	67.1	52.1
*E. coli*	95.6	95.3	93.4	84.2	92.0	91.8	95.1	95.9
*B. subtilis*	37.4	37.9	17.6	67.7	26.3	10.0	61.2	26.8
*V. atlanticus*	65.6	61.8	37.5	4.7	9.8	16.2	44.6	31.4
Total alignment (kbp)	9535	9119	6954	7082	5893	5556	9229	7270
Largest alignment (kbp):								
*E. coli*	223.9	258.4	155.5	68.7	107.4	78.4	173.6	217.9
*B. subtilis*	61.0	17.4	23.4	39.2	25.6	11.8	33.2	18.2
*V. atlanticus*	64.6	45.4	45.6	12.6	15.5	9.9	24.8	29.7
Unaligned fraction	1.1	1.2	1.3	1.3	1.6	1.6	1.1	1.2
Total length (kbp)	9642	9232	7048	7175	5991	5646	9328	7360
#Contigs (≥1000 bp)	1147	1280	791	1044	692	736	1287	825
#Contigs (≥10,000 bp)	150	124	127	183	122	129	160	84
Largest contig (kbp)	233.9	259.0	160.4	68.7	132.8	89.4	173.6	218.0
N50	26,721	16,815	27,496	11,101	22,467	18,168	15,283	47,972
Mean sequencing coverage	40.0	35.6	45.2	43.3	49.0	48.8	40.2	48.1
Standard deviation of sequencing cover	56.8	44.9	57.6	69.2	66.3	67.4	57.9	59.2
Number of protein-coding genes	8872	8491	6473	7043	5720	5276	8658	6772
Average protein length (aa)	277	267	280	262	272	273	271	269

**Table 3:** Overview of invertebrate samples used in this study, with taxonomic inference based on either 28S rRNA and CO1 genes or on established morphological criteria.

Sample	Origin	Taxonomic inference
A	Sponge fragment	Order Poecilosclerida *
B	Sponge	Halichondria panicea *
C	Sponge	Order Poecilosclerida *
D	Sponge fragment	Family Myxillidae *
E	Bryozoa	*Alcyonidium gelatinosum*
F	Sponge	Genus *Myxilla* *

* Lowest consensus taxonomic rank between 28S rRNA and CO1 gene sequences based on BLAST searches (NCBI nr/nt database).

**Table 4:** Summary of results from the assembly of marine invertebrate microbiome MDA products. The estimated number of cells in each sample before MDA is reported together with assembly statistics. The mean sequencing coverages and their standard deviations, as calculated by aligning the reads to the assemblies, are also shown. The total length and number of contigs are shown for contigs >1000 bp.

Sample	Estimated # Cells	Total Length (kbp)	Largest Contig (kbp)	N50	# Contigs	# Contigs (>10,000 bp)	Sequencing Coverage	SD
A1	2	5867	34.3	3992	1717	78	59.3	198.5
A2	2	8179	27.2	4005	2393	99	60.8	197.9
A3	170	31,262	68.6	1715	11,673	437	8.0	11.9
A4	170	33,557	168.3	2011	10,984	480	8.4	14.0
B1	75	29,443	106.9	1815	10,708	396	5.9	26.8
B2	75	30,326	102.2	1794	11,256	387	6.4	22.0
B3	375	24,807	124.5	1533	10,002	226	5.6	23.8
C1	26	8588	41.0	3851	2483	156	34.6	99.4
C2	54	17,962	89.4	2264	5830	277	12.5	35.4
D1	7	19,116	66.1	2093	6617	278	9.2	42.7
E1	2	5193	30.1	3421	1642	54	43.6	150.7
E2	425	15,303	40.2	1205	6246	161	6.4	25.1
E3	850	4520	29.8	3655	1408	47	107.0	292.4
F1	2	24,272	98.6	3378	7056	423	9.6	19.5

**Table 5:** The number of genes annotated with EC-numbers corresponding to a selection of enzymes with potential biotechnological relevance.

		A1	A2	A3	A4	B1	B2	B3	C1	C2	D1	E1	E2	E3	F1	Total
Triacylglycerol lipase	EC 3.1.1.3			1	2			1		1	1		1		1	8
Protease	EC 3.4	34	42	136	169	125	129	95	32	70	72	27	75	20	121	1147
Chitinase	EC 3.2.1.14			1		1	1					1				4
Alpha-amylase	EC 3.2.1.1					3	4	2								9
Cellulase	EC 3.2.1.4					1	1									2
Lysozyme	EC 3.2.1.17			1		5	5	4							2	17
Xylanase	EC 3.2.1.8/32					4	4	2			1					11
Xylosidase	EC 3.2.1.37/72					2	2	3								7
DNA ligase (ATP)	EC 6.5.1.1	1	1	7	10	8	12	8	2	7	8		1		3	68
DNA ligase (NAD+)	EC 6.5.1.2	3	2	13	24	14	9	12	2	7	5	2	8	2	9	112
RNA 3’-terminal-phosphate cyclase (ATP)	EC 6.5.1.4	1		1	1								2			5

## Data Availability

The sequence data generated in this study are available at the European Nucleotide Archive (ENA) (https://www.ebi.ac.uk/ena/browser/home). The study can be found under the accession PRJEB57785 or secondary accession ERP142786. The raw reads can be found under the accessions ERR10555228 to ERR10555241 (marine invertebrate microbiomes) and ERR10561858 to ERR10561875 (reference strain genomes).
